# An Innovative Technique to Immediately Load a Severely Atrophic Maxilla Without Zygomatic Dental Implants Using the 3B-TB Protocol

**DOI:** 10.7759/cureus.93170

**Published:** 2025-09-25

**Authors:** Murugavel Chandrasekaran, Vivek Pandian Chellapandi, Deenadayalan Narasimman, Deepak Abraham Pandyan, Balamurugan Rajendran

**Affiliations:** 1 Dentistry, Oral Surgery, Oral Implantology, Full Arch Institute, Chennai, IND; 2 Dentistry, Oral Surgery, Oral Implantology, Dr. Vivek’s Msrams Dentistry Private Limited, Chennai, IND; 3 Dentistry, Oral Surgery, Oral Implantology, Clinical Director, Best Dental Clinic, Chennai, IND; 4 Oral and Maxillofacial Surgery, Madha Dental College and Hospital, Chennai, IND; 5 Dentistry, Oral Surgery, Oral Implantology, RYA Cosmo Foundation Hospital, Chennai, IND

**Keywords:** 3b-tb protocol, atrophic maxilla, compressive multiunit implants, no zygoma approach, palatal roll over flap method

## Abstract

Zygomatic dental implants are a standard treatment option in the management of severe atrophic maxilla with a good survival rate. However, zygomatic implants have potential complications, drawbacks, limitations, and risks of failure. The 3B-TB protocol is an innovative technique to immediately load a severely atrophic maxilla without placing zygomatic implants. The aim of this technical note is to avoid zygomatic implants and their associated complications, and elaborates on the management of severely atrophic maxillae using the 3B-TB protocol of implant placement and prosthetic restoration. The 3B-TB protocol was critically planned by implementing a combination of key mechanical and surgical principles for full mouth rehabilitation. Rehabilitation of severely atrophic maxilla by this protocol does not require an implant support in Bedrossian zone 3, 2 (molar and premolar region). Long bending moments and excess micro-movements at the bone implant interface are reduced, which ultimately facilitates immediate loading, safe osseointegration, and long-term stability. Surgical techniques, combined with uniquely designed single-piece compressive multi-unit implants, support engagement even in unfavorable pterygomaxillary areas, with 2-4mm of bone width, and 2-8mm of bone height. The 3B-TB protocol is a successful and promising alternative treatment choice to other available treatment modalities like zygomatic implants and bone grafts in the management of severe atrophied maxilla with minimal to no complications.

## Introduction

Rehabilitation of the atrophic maxilla with dental implants presents specific challenges for clinicians. The atrophy mainly results from two factors: increased pneumatization of the maxillary sinus in both anterior and posterior directions and bone resorption in both vertical and horizontal dimensions following tooth extraction [1,2]. Bone resorption is further triggered by pre-existing periodontal disease, periapical infection, long-term denture wear, and systemic patient factors [3,4]. The resultant severe maxillary atrophy complicates conventional implant treatment. To address the challenges associated with maxillary atrophy, regenerative techniques such as bone augmentation and sinus lift procedures, with or without the use of bone grafts, are performed [1]. However, these augmentation techniques have several drawbacks, including increased morbidity, longer treatment duration, higher costs, greater risk, and a potential reduction in patient quality of life [5]. As an alternative to bone augmentation procedures and sinus lifts, zygomatic implants were introduced by Branemark in the late 1980s [6,7].

The use of zygomatic implants has increased significantly in recent years, supported by documented high survival rates [6]. They serve as a reliable alternative to traditional augmentation procedures by eliminating the need for bone grafts, reducing the number of implants required, lowering the treatment costs, and shortening the treatment time. One of the key advantages of zygomatic implants is the possibility of immediate loading. A recent systematic review reported a five-year success rate of 97.6% for zygomatic implants [8]. However, due to the extended length of these implants and the proximity of critical anatomical structures along their path, there is a risk of potential complications. These may include invasion of the orbit and infratemporal fossa, chronic maxillary sinusitis, oral vestibular dehiscence, peri-zygomatic infections, oroantral fistula, implant fracture, and other related issues [9]. Therefore, it should be performed only by clinicians with appropriate training and experience to minimize complications and failures [10].

There has been a recent increase in interest among clinicians to avoid zygomatic implants whenever possible. Additionally, patients fearful about the invasive nature of zygomatic implants actively seek alternative solutions for getting immediate fixed teeth replacements without zygomatic implants. The 3B-TB protocol has been proposed for immediate functional load of atrophic edentulous maxilla without using zygomatic implants. This technical note elaborates on the biomechanical principles, surgical principles, and uniquely designed single-piece compressive multi-unit implants used in this protocol.

## Technical report

**Figure 1:**
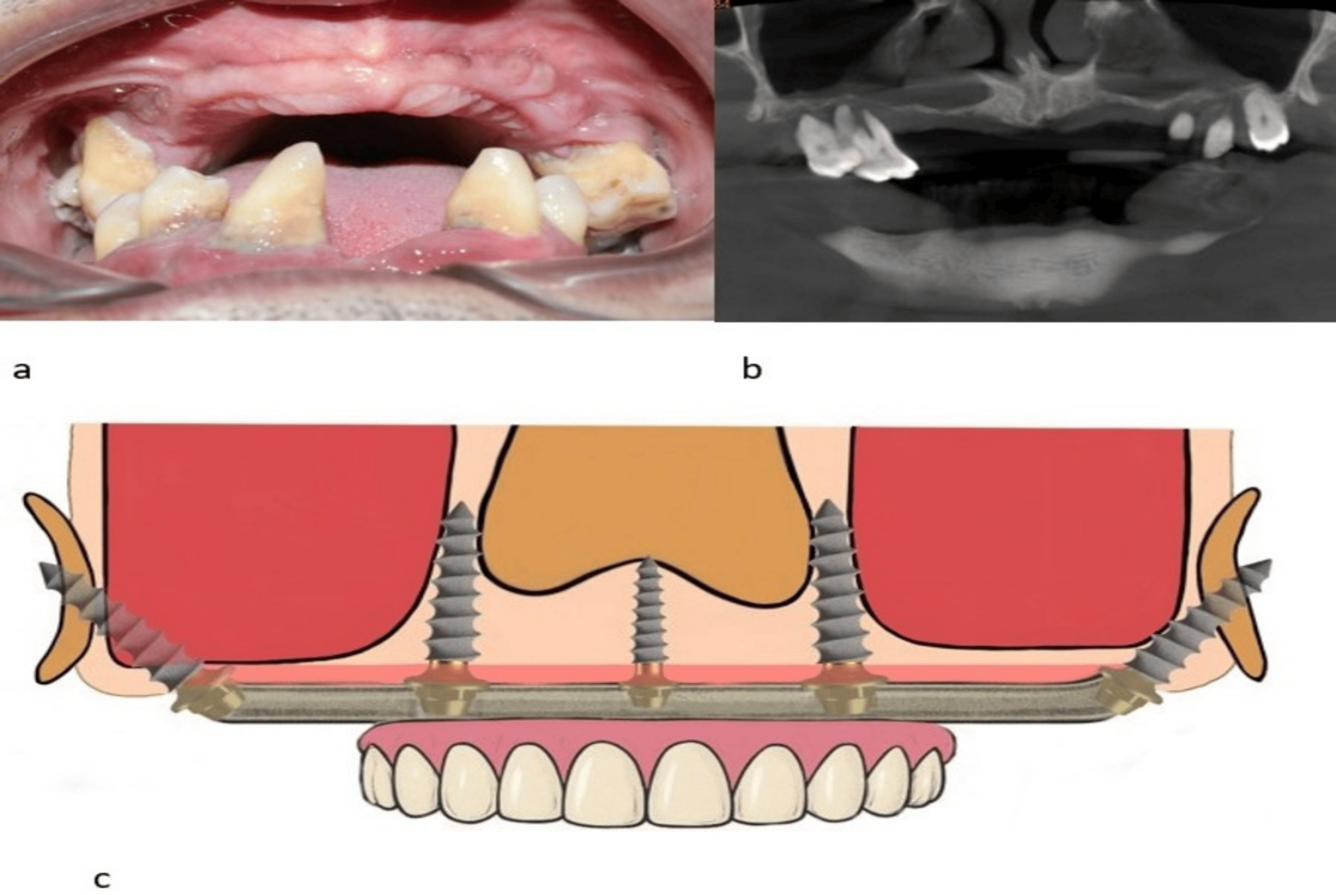
a: Intraoral image showing severely atrophic maxilla. b: Preoperative orthopantamograph showing severely atrophic maxilla. c: Pictorial representation of implant placement using the 3B-TB protocol The images submitted are original and published under a Creative Commons License

This technical note presents a 52-year-old man with a history of chronic gum disease for more than 25 years who presented to our centre for full mouth implant rehabilitation. He is a known diabetic and under medication. The patient had already visited numerous clinics and had been advised of zygomatic implant treatment. However, he was seeking fixed tooth replacement without the use of zygomatic implants. Clinical examination revealed few existing periodontally compromised teeth in both jaws. Cone beam computed tomography (CBCT) examination revealed severe maxillary atrophy with sinus pneumatization extending anteriorly till the canine region and posteriorly extending into the tuberosity bone on both sides (Figure 1A, 1B). The bone width in the canine region was 4-5mm, and the bone height in the right and left canine regions was 10mm and 8mm, respectively. The treatment modality was planned for immediate full arch rehabilitation with single-piece compressive multi-unit implants using the surgical and biomechanical principles of the 3B-TB protocol under local anesthesia (Figure 1C).

Steps in the 3B-TB protocol

Steps in the 3B-TB protocol include engagement of uniquely designed single-piece compressive multi-unit implants in the region of the pterygomaxillary buttress, nasomaxillary buttress, and nasopalatine buttress. Conventional implants may also be preferred if the available buccolingual width is 4mm or greater and available bone height is 8mm or greater in zone 1 [11]. Implants were splinted with a passively fitting, screw retained titanium bar. This titanium bar helps with optimal stress distribution during mastication. Prosthetic teeth with a low elastic modulus were used, which helps for optimal stress absorption during mastication.

Principles of the 3B-TB protocol

The 3B-TB protocol was implemented based on a combination of key biomechanical and surgical principles for full mouth rehabilitation.

Biomechanical Principles

The biomechanical principles establish the core foundation of the 3B-TB protocol for avoiding zygomatic implants. Generally, zygomatic implants are indicated when conventional implants cannot be placed in the premolar and molar region due to sinus expansion or insufficient bone width. However, according to the biomechanical principles of the 3B-TB protocol, this kind of atrophic situation can be rehabilitated by placing implants only in the pterygomaxillary region and placing three to four implants in the premaxillary region (from canine to canine), provided that proper suprastructure and substructure are selected. The correct selection of supra- and substructure will help to reduce the excess micro-movements within the bone implant interface, enabling immediate loading, safer osseointegration and, long-term stability.

Considerations for the Selection of Proper Suprastructure and Substructure

Selection of proper supra structure (for better stress absorption): Selecting prosthetic teeth with a low elastic modulus is recommended, as they exhibit good stress absorption properties. This stress-dampening property will reduce the forces transmitted to the bone and help to minimize the excess micro-movements at the bone implant interface. 3D printed resin bridges or milled poly-methyl methacrylate (PMMA) bridges are recommended for a provisional full arch prosthesis, which will be delivered within four to five days of implant placement. Composite bridges or Graphene (G-Cam) bridges are recommended for a durable prosthesis, which will be delivered after six months. The elastic modulus values for all the above-mentioned materials are between 2700 mpa to 3200 mpa.

Selection of proper substructure (for better stress distribution): Selecting a substructure with a high elastic modulus is recommended, as it exhibits better stress distribution properties and higher resistance to deformation. These properties will reduce the forces transmitted to the bone and help to minimize the excess micro-movements at the bone implant interface. Titanium bar is recommended as the substructure material in the 3B-TB protocol for better splinting of implants and stress distribution. Titanium bar has key advantages, including high flexural strength, low weight, no galvanic corrosion and biocompatibility. The elastic modulus of titanium is 1,10,000 mpa.

Surgical Principles

The surgical principles of the 3B-TB protocol provide guidance over the selection of implant location and number, type of implant, pterygoid approach, palatal techniques, implant diameter, length, and soft tissue closure method. By following these six surgical principles, implants can be placed even in an unfavorable pterygomaxillary region, even if the available bone width is 2-4mm and bone height is 2-8mm.

Selection of location and number of implants: Three anatomical buttresses of the maxilla are selected for implant placement, namely the pterygomaxillary buttress, the nasomaxillary buttress, and the nasopalatine buttress. Since implants are engaged in three buttresses in the maxilla and splinted with a titanium bar, this protocol is named the 3B-TB protocol. Based on the location and number of implants, there are two patterns of implant placements: 3BTB-6 and 3B-TB-5.

3B-TB-6 represents placement of two implants in the pterygomaxillary buttress on either side, two implants in the nasomaxillary buttress on either side, and two implants in the nasopalatine buttress area on either side of the nasopalatine canal (areas of lateral incisors). 3B-TB-6 is indicated in two circumstances: When the inter sinus width is sufficient (distance between the right and left maxillary sinus) for the placement of four implants. When the available bone in the lateral incisor area is favorable for implant placement.

3B-TB-5 represents placement of two implants in the pterygomaxillary buttress on either side, two implants in the nasomaxillary buttress on both sides, and one implant in the nasopalatine canal or labial to nasopalatine canal. 3B-TB-5 is indicated in two circumstances: when the inter sinus width is less due to expansion of the maxillary sinus extending up to the canine region. When the areas of the lateral incisor are completely unfavorable for implant placement.

**Figure 2:**
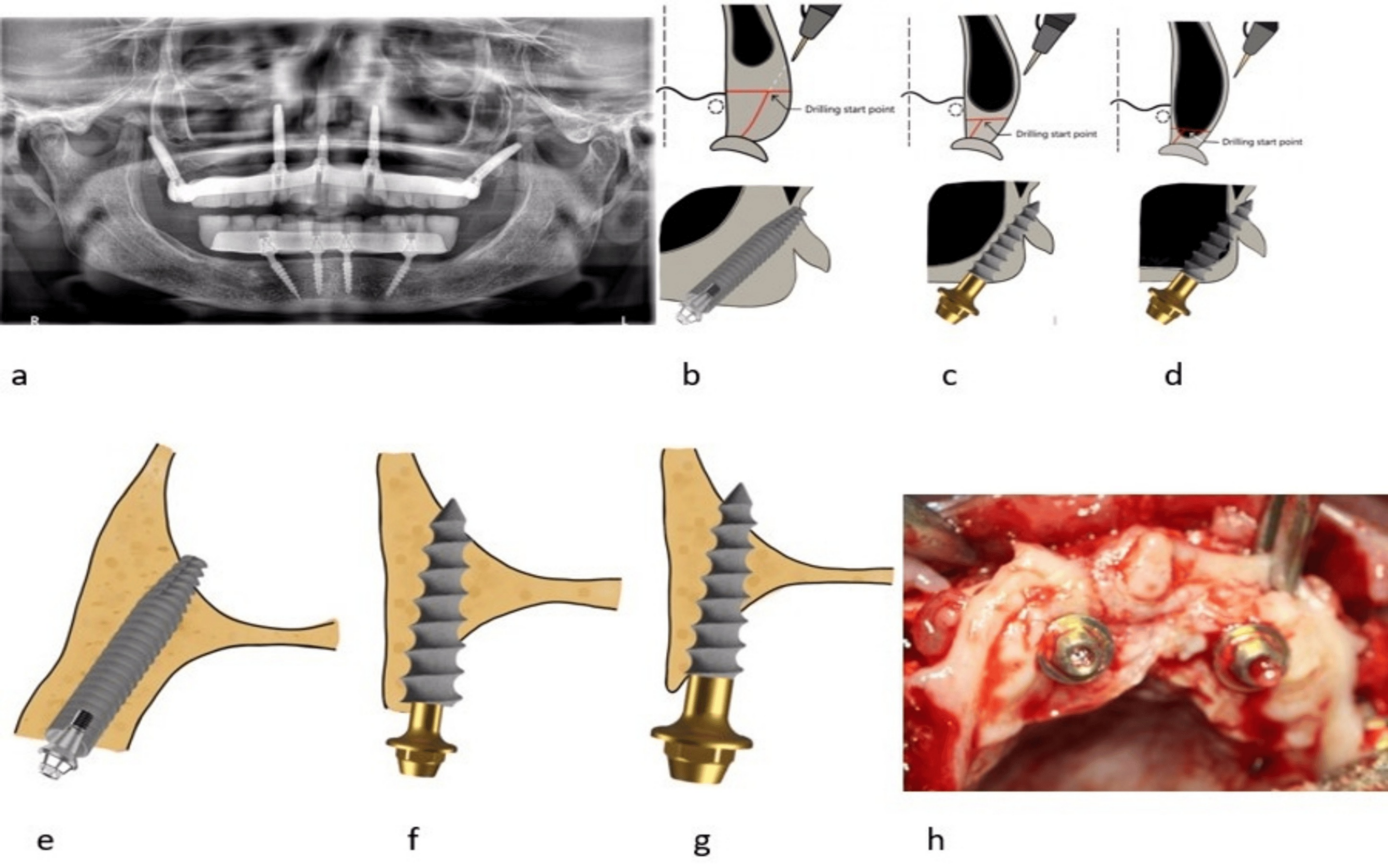
a: Orthopantamograph showing implant placement using Vanderlim’s transnasal approach by engaging the inferior conchal bone bilaterally along with bilateral pterygoid implants and midline nasopalatine implant. b: Pictorial representation of implant placement using regular pterygoid approach. c: Pictorial representation of implant placement using distalised pterygoid approach. d: Pictorial representation of implant placement using trans-sinus pterygoid approach. e: Pictorial representation of implant placement using regular crestal technique. f: Pictorial representation of implant placement using palatal crestal technique. g: Pictorial representation of implant placement using palatal to palatal crestal technique. h: Intraoperative image showing protection of thin labial bone using roll-over flap technique The images submitted are original and published under a Creative Commons License

In a severe atrophic situation, when naso-maxillary buttress implants cannot be placed or premaxillary bone height is only 2mm-5mm, Vanderlim’s transnasal implants can be placed by engaging the inferior conchal bone bilaterally along with bilateral pterygoid implants and midline nasopalatine implant (Figure 2A) [12].

Selection of type of implants: Two types of implants can be utilised in the 3B-TB protocol, namely conventional implants and single-piece compressive multiunit implant (CMU implants, BioLine Dental Implant Systems Ltd., Migdal Tefen, Israel). Conventional implants are indicated in the following four circumstances: when there is sufficient tuberosity bone available, pterygoid anatomic radiographic prediction (PARP) Type 1, 2 [13] to guide conventional implant placement towards the pterygoid area, conventional implants can be used; when the available bone width is 6-8mm or more in zone 1 (premaxilla) and zone 2 (premolars); when the available bone height is 8mm or more, with no labial defects in zone 1, zone 2; and when the available bone height is less than 5mm, long conventional implants can also be used as trans-nasal implants. Single-piece compressive multiunit (CMU) implants are indicated in three circumstances: when there is insufficient tuberosity bone available due to sinus expansion (PARP Type 3, 4) [13]; when the available bone width is only 2-4mm in zone 1 and zone 2; and when the available bone height is only 6-8mm in zone 1 and zone 2.

Approach for pterygoid implant placement: Three approaches are used to place an implant in the pterygomaxillary buttress area: the regular pterygoid approach, the distalised pterygoid approach, and the trans-sinus pterygoid approach. Approach type depends on the severity of distal maxillary sinus expansion into the maxillary tuberosity. The greater the distal maxillary sinus expansion, the less the available maxillary tuberosity bone to guide the drilling and implant placement towards the pterygoid area. A regular pterygoid approach is used when sufficient tuberosity bone is available (PARP 1 and 2) to guide implant placement. Distalised pterygoid and trans-sinus approach are used when insufficient tuberosity bone is available due to sinus expansion (PARP 3 and 4).

Regular pterygoid approach (Figure 2B) is used in cases of PARP 1, 2 type, where there is enough bone in the tuberosity to guide drilling and implant insertion. The crestal drilling point is initiated approximately 10mm or more in front of the distal end of the tuberosity. The conventional pterygoid implants are preferred for this approach.

The distalised pterygoid approach (Figure 2C) is used in the PARP 3 pterygoid type. Considering the more distal expansion of the maxillary sinus into the tuberosity, the crestal drilling starting point is made approximately 6-8mm in front of the distal end of the tuberosity. The implant type preferred with the distalised pterygoid approach is either conventional pterygoid implants (diameter 3.5mm or less) or CMU implants. However, using conventional implants carries the risk of crushing the tuberosity during implant insertion or implant misdirection towards the sinus.

The trans-sinus pterygoid approach (Figure 2D) is used in the PARP 4 type pterygoid. Considering the very severe distal expansion into the maxillary tuberosity, leaving very limited bone in the tuberosity, the crestal drilling starting point is made 4-5mm in front of the distal end of the tuberosity. Implant type preferred with the trans-sinus pterygoid approach is either conventional pterygoid implants (diameter 3.5mm or less) or CMU implants. However, using conventional implants carries the risk of crushing the tuberosity during insertion or implant misdirection towards the sinus. If proper placement is achieved, the implant will traverse through the sinus along the distal sinus wall to engage the pterygoid area.

Using the distalised pterygoid approach and the trans-sinus pterygoid approach with an appropriate implant design (≤3.5mm conventional pterygoid implant or CMU implants) helps to achieve adequate stability, even in unfavorable PARP types 3 and 4. Using CMU implants in these two challenging situations is more reliable and predictable than using conventional implants. The risk of the implant not engaging or slipping into the maxillary sinus and crushing the tuberosity bone is minimal with CMU implants. A distalised pterygoid approach or a Trans-sinus pterygoid approach, in combination with CMU implants, will increase the success rate of pterygoid implant placement and reduce the need for posterior zygomatic implants.

Techniques for implant placement in zone 1 and zone 2: Three techniques are used, namely, crestal technique, palatal crestal technique, and palatal to palatal crestal technique. In the regular crestal technique (Figure 2E), drilling is started in the midcrestal area. This technique is used when the buccopalatal bone width is sufficient to place a conventional implant. However, the regular crestal technique cannot be considered when the bone width is insufficient.

The palatal crestal technique (Figure 2F) is used when the available buccopalatal width is only 5-6mm. The drilling is initiated at the palatal crestal area instead of the mid crestal area [14]. With this approach, a minimum of 2mm of bone remains intact on the labial aspect of the implant. After implant placement, the implant threads may get exposed crestally on the palatal side. However, thick palatal tissue protects the exposed implant threads. Both CMU implants and smaller-diameter conventional implants (≤ 3.5mm) are preferred in this approach.

Palatal to palatal crestal technique (Figure 2G) is used when the buccopalatal width is only 2mm-4mm. The drilling is initiated palatally to the palatal crest entirely on the palatal side. In this way, 1.5-2mm of labial bone is protected, and the exposed implant threads on the palatal crestal side are protected by the thick palatal tissue. There is no need for any bone grafts to cover the exposed palatal implant threads. CMU implants are preferred for this technique over conventional implants. Employing these two techniques, namely the palatal crestal technique and the palatal to palatal crestal technique with CMU implants, will help to overcome the deficiencies in buccopalatal width and the presence of labial bone defects in an efficient manner. CMU implants have a tapered body design and thin apical diameter, which helps to get engagement in the nasal floor without fracturing the thin labial bone.

Selection of implant length: When the available bone height is only 6-8mm in zones 1 and 2, 8-10mm length CMU implants can be used with 1-2mm of implant tip extending into the nasal floor. CMU implants are preferred when the bone is deficient both in width and height. When the available bone height is only 2-6mm in zone 1 and zone 2, longer (20-25mm) implants can be used in the canine area trans-nasally to engage the inferior conchal bone by Vanderlim’s trans-nasal implant technique [13].

Soft tissue closure technique: By using palatal techniques, even 1.5-2mm bone width areas can be rehabilitated with dental implants. However, maintenance of thin labial bone is crucial for the long-term stability and success of implants, which can be achieved by using the palatal roll-over flap method. This technique brings the thick palatal gingiva to the labial side, thereby protecting the thin labial bone from resorption (Figure 2H).

Surgical phase

The management of severe atrophic maxillae was surgically delineated using the 3B-TB protocol of dental implant placement and prosthetic restoration. Informed patient written consent was obtained prior to surgery. The patient was pre-medicated with amoxicillin 500mg capsules, ibuprofen 400mg tablets, prednisolone 20mg tablets, and alprazolam 0.5mg tablets one hour before the commencement of surgery. The surgical procedure was performed under an aseptic environment. Intraoral 0.2% chlorhexidine irrigation was done. Optra Gate was placed to have better peri-oral tissue retraction, visibility, and accessibility of the operating site. Local anaesthesia was administered with 2% lignocaine hydrochloride and 1:80,000 concentration of adrenaline. The basic sequence followed was pterygoid implant placement bilaterally with the open flap method, flap elevation in zone 2, 1 followed by alveoloplasty, pencil markings for nasomaxillary buttress implant and nasopalatine buttress implants, drilling and implant placement, wound debridement and suturing (Video 1).

**Video 1:** 3B-TB surgical procedure

Pterygomaxillary Buttress Implant Placement

The pterygomaxillary buttress implant was placed first, followed by the other implant placement. This “pterygoid-first approach” has the following advantages: better patient cooperation and good mouth opening at the beginning of surgery helps the operator to place pterygoid implants without any difficulty, and also, if pterygoid implants were attempted first, the need for zygomatic implants can be decided and planned at the initial stage of surgery itself.

Crestal incision was made with BP blade no 15 from the distal end of the maxillary tuberosity till the first molar region, and a buccal vertical releasing incision in first molar region. The mucoperiosteal flap was elevated to expose the tuberosity buccally and distally till the hamular notch. The palatal flap was minimally reflected (3-4mm) to protect the greater palatine foramen and artery. Flap reflection and exposure of the medial and distal-most end of the tuberosity were done, which is the key anatomical landmark guiding the operator to place the implants accurately towards the pyramidal and pterygoid process. The medial and distal-most end of the tuberosity was retracted with the blunt end of a periosteal elevator, which also acts as a guide during drilling to achieve the correct medial angulation.

**Figure 3:**
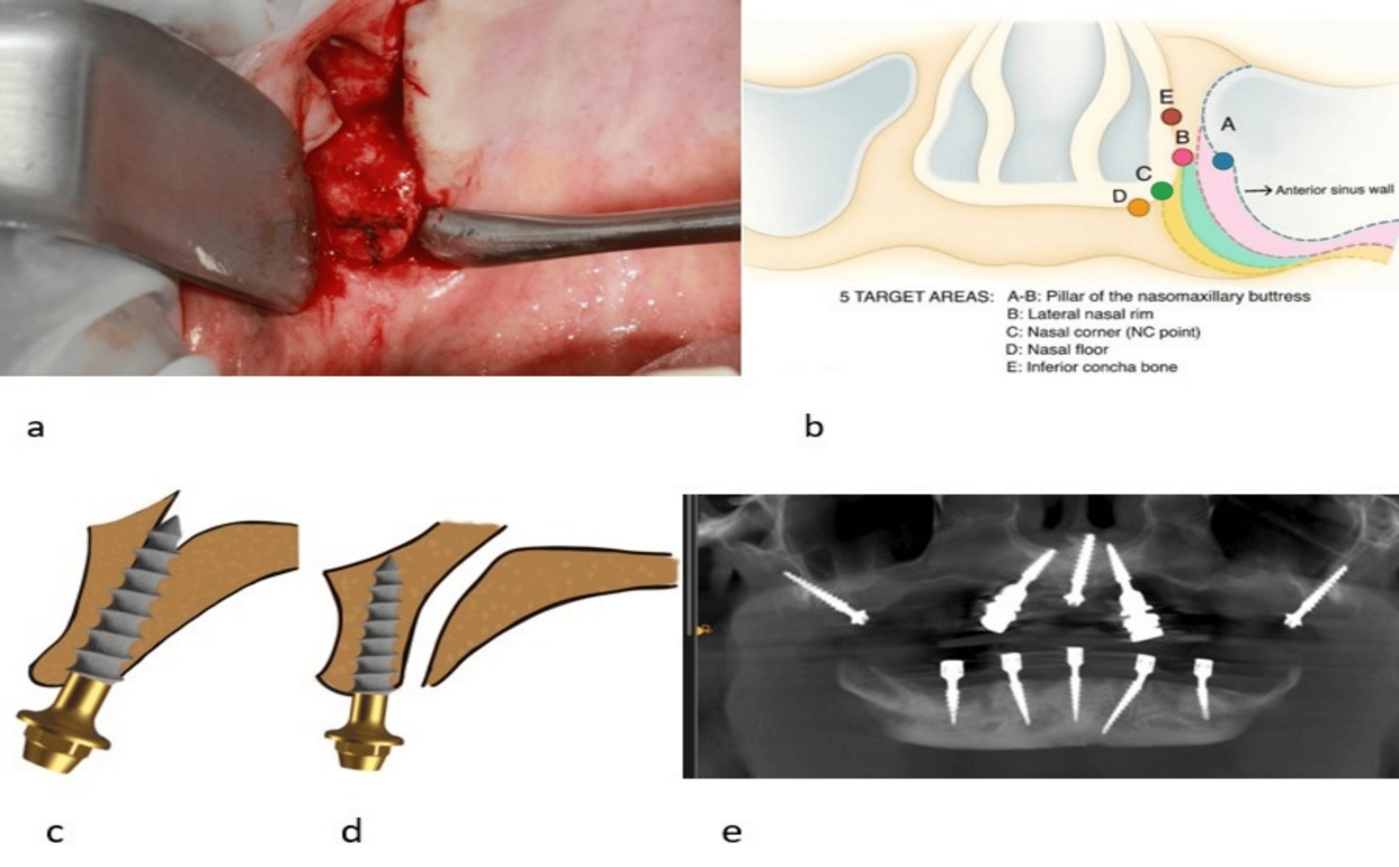
a: Intraoperative image showing “T” marking for pterygomaxillary buttress implant. b: Target areas for Implant placement in the nasomaxillary buttress area based on the severity of sinus expansion. c: Pictorial representation of implant placement into the nasopalatine canal. d: Pictorial representation of implant placement anterior to the nasopalatine canal. e: Postoperative orthopantamograph showing implant placement in the severely atrophic maxilla using the 3B-TB protocol The images submitted are original and published under a Creative Commons License

A “T” marking was placed over the crestal aspect of the maxillary tuberosity with a pencil (Figure 3A). The horizontal marking was drawn buccopalatally in the alveolar crest, 4-12mm in front of the distal end of the tuberosity. However, this distance will be determined by the severity of the posterior sinus expansion. The greater the posterior sinus expansion, the more distal the horizontal markings will be placed. From the horizontal marking, a vertical marking of “T” was drawn to the distal and medial-most end of the tuberosity. The junction of the vertical and horizontal marking guides the initial entry point of the drill, and the vertical marking will guide the medial angulation of the drilling towards the pyramidal and pterygoid process. In cases of PARP types 1 and 2, surgical drilling was initiated 10-12mm mesial to the distal end of the tuberosity, which is represented by a regular pterygoid approach. Whereas, in the PARP 3 type, the drilling was initiated 6-8mm mesial to the distal end of the tuberosity, and in the PARP 4 type, the drilling was initiated 4-5mm mesial to the distal end of the tuberosity. The vertical marking guides the drill towards the pyramidal process/pterygoid process, which is the pterygoid pillar composed of good-quality cortical bone. These horizontal and vertical markings, known as the "T mark", provide a clear visual reference for the operator during drilling to enter the correct target area, thereby increasing accuracy and reducing the duration of the procedure.

Pilot drilling was performed using a surgical straight handpiece with a “T” marking as guidance. After 5-6mm of surgical drilling, an intraoral periapical radiograph (IOPA) was taken to verify the drill angulation in the supero-inferior direction. Any correction in drilling angulation, superior-inferiorly, can be made at this stage. Drilling in the inferior direction may enter the non-fusion zone below the hamular notch, which is composed of poor-quality bone. While drilling in the superior direction may enter the maxillary sinus. At this stage, IOPA helps to avoid errors in the superior-inferior direction. Hence, a combination of “T” marking for anatomical guidance and IOPA for radiographic guidance helps to achieve accuracy in three dimensions: drilling starting point, medial angulation, and superior-inferior angulation. Further pilot drilling was continued to assess the resistance of the pterygoid pillar. After the resistance, a drop will be felt as the drill exits the pterygoid pillar. Appropriate implant length was selected based on the drill drop. After completion of pilot drilling, a 2mm twist drill can be used if the bone quality is too dense. Since the CMU implant apex diameter is only 1.8-2mm, there is no need to use the 2mm twist drill. CMU implants can be inserted after the single drill. However, if conventional pterygoid implants are planned, sequential drilling must be carried out.

Nasomaxillary Buttress Implant Placement in Zones 1 and 2

The nasomaxillary buttress is the pillar of bone present between the lateral nasal rim and the anterior wall of the maxillary sinus. The width and thickness of this buttress vary widely. The width of the buttress decreases as the sinus expands anteriorly. In severe sinus expansion, the width and thickness of the nasomaxillary buttress are insufficient to place implants.

Hence, five options are available for implant apex engagement in the nasomaxillary buttress area, depending on the severity of sinus expansion. They are the naso maxillary buttress proper, lateral nasal rim, nasal corner, nasal floor, and inferior conchal bone (Figure 3B). Depending on the degree of sinus expansion in the crestal region, three options are available for implant positioning at the crestal area, which include the second premolar, first premolar and canine.

The crestal incision was made from molar to molar bilaterally, and the mucoperiosteum was reflected to expose the lateral maxillary wall, nasomaxillary buttress, lateral nasal rim, and nasal floor. Care was taken not to injure the infraorbital nerves and nasal floor membrane. The palatal flap was elevated up to 5-6mm, and the content of the nasopalatine canal area was severed with the BP blade no 15. A tie suture was placed to retract the palatal flap during surgery. Alveoloplasty, if required, was performed using bone rongeurs and bone trimmers. Implant locations and their paths were finalized and marked with a pencil, and surgical drilling was initiated with a pilot drill, followed by a 2mm twist drill. An appropriate implant length of single-piece CMU was selected and installed in a tilted position. If the bone width is 4mm or greater, conventional implants can be utilized, and suitable multiunit abutments are preferred to correct the angulation of conventional implant. If the bone width is less than 4mm, the palatal technique is used for drilling and implant placement.

Nasopalatine Buttress Implant Placement

Three surgical methods are available for implant placement in the nasopalatine buttress. Method 1 represents placement of two implants on either side of the nasopalatine canal when there is sufficient bone width and height. Method 2 represents placement of one implant in the nasopalatine canal (Figure 3C). Method 3 represents placement of one implant labial to the nasopalatine canal (Figure 3D).

Methods 2 and 3 are preferred when the bone quality in the incisor area is unfavorable or the inter-sinus width is insufficient to accommodate four implants in zone 1. Once the implants are placed, relevant multiunit abutments are chosen if conventional implants are placed. If single-piece CMU implants are selected, mild bending up to 15-20 degrees may be considered to correct angulation. Once the implants are placed in the desired position (Figure 3E), saline irrigation was done, and sharp bony margins were smoothened. A palatal roll-over flap technique was used if an excess palatal flap was present. This technique protects 1.5-2mm of thin labial bone over implants by bringing the palatal keratinized gingiva to the labial side. A small incision of 4-5mm in length was made in the excess palatal flap at a corresponding location of multiunit abutment, and the palatal flap was rolled over the multiunit abutments to approximate with the labial flap. Final suturing was performed with 3-0 Vicryl sutures. Postoperative instructions and medications were prescribed with antibiotics and analgesics for five days. A gentle mouthwash with 0.2% chlorhexidine was recommended for two weeks postoperatively (Table 1).

**Table 1:** Differences in the implant design and functional outcomes between single piece compressive multiunit implants (CMU) and conventional implants

Compressive Multiunit Implants (CMU)	Conventional Implants
Implant body and multiunit abutment are in one piece	Implant body and multiunit abutments are available in two separate pieces connected with a screw
Tapered implant body design. Tapering feature is present from the crestal 1^st^ thread to the apex	Both tapered and non-tapered Implant design are available. The crestal part of the implant is almost parallel
Tapered implant body design with 1.8-2 mm apex allows easy insertion in thin ridges (up to 1.5mm). Less risk of labial bone fractures	Due to the parallel design of the coronal part of the implant and larger apical diameter, it is difficult to use in 1.5-2mm thin ridges. More risk of labial bone fracture during implant insertion
1.8mm apical diameter is possible even in shorter length implants (8mm). This helps to place implants in bone that is deficient in both height and width	Short apical diameter is not possible with shorter length (8mm, 10mm) conventional implants. The conventional short implants cannot be used in cases of 6-8mm height, where the bone width is also deficient
Easy to get bicortical engagement with thin tapered apex with minimal drilling. Less risk of implant spinning in the osteotomy site without cortical engagement	Risk of implant not engaging bicortically and the implant spinning is more if the drilling is insufficient
Absence of multiunit abutment/implant connection. So no risk of micro gap and micro movement. And no risk of screw loosening and screw fracture in the multiunit abutment/implant junction	Presence of multiunit abutment/implant connection. Risk of micro gap and micro movement is present, if manufacturer instructions are not properly followed. Risk of screw loosening and screw fracturing in multiunit abutment/implant junction
Inbuilt platform switching due to thin neck (2.2 mm diameter). This design promotes better gingival health in peri-implant area	Platform switching feature is available based on implant brands and selection of appropriate multiunit abutments
Implant angulation correction is possible by five methods: Proper prosthetic and surgical planning before implant placement Avoiding more than 30-degree angulated placement Bending of multiunit abutment-implant is possible up to 20 degrees provided sufficient primary stability is achieved When bone quality is poor or short implants are planned, bending of CMU implants is not recommended. So CMU implants are placed either axially or tilted only up to 15-20 degrees. By using pre-angulated (17 degree, 30 degree) CMU implants	Implant angulation correction is possible by two methods: Proper prosthetic and surgical planning before implant placement by using angulated multiunit abutments (0-60 degrees)
Risk of implant neck fracture if excess torque is applied during insertion. However loosening of implant carrier occurs when excess torque is applied to the implant. This acts as a fail-safe mechanism to prevent implant neck fractures	Risk of implant crestal part fracture and implant carrier fracture is possible, if excess torque is applied during implant insertion
Screw retained prosthetic options are possible	Screw retained prosthetic options are possible
Damage to prosthetic screw channel or prosthetic screw fracture in multiunit abutment is difficult to resolve. However the chances of this complication are very rare.	Any damage at multiunit abutment level can be solved by replacing with new multiunit abutment

Prosthetic phase

**Figure 4:**
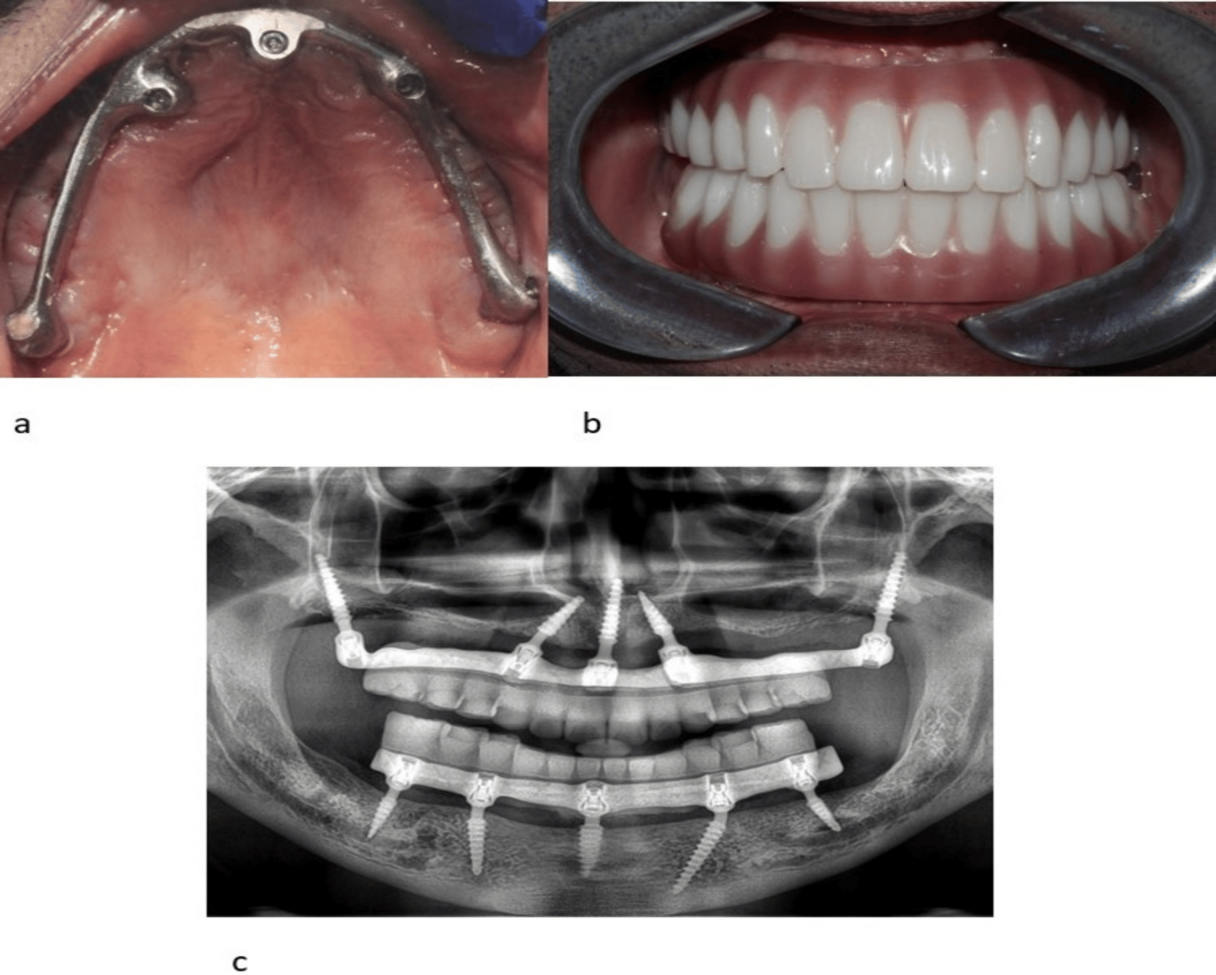
a: Intraoral image showing titanium bar placement. b: Intraoral image showing implant supported prosthetic replacement using 3B-TB protocol. c: Two years follow-up postoperative orthopantamograph showing implant supported prosthetic replacement using the 3B-TB protocol

On the same day after the implant placement, digital scan bodies were fixed over the implants, and intraoral scanning was performed with a 3shape scanner. On day 2, the jaw relation was recorded for the patient using Alu wax bite rims. Bite rim scans and jaw relation scans were taken and incorporated into the Exocad software along with the implant scan. Prototype teeth were designed using Exocad software, and the I Bar design was performed using Blender software. On day 3, the printed prototype trial was checked on the patient to ensure accurate fit and aesthetics, and assessed for vertical dimension, centric relation, and occlusion. After obtaining satisfactory trial approval from the patient, I-bar was milled in a CAD/CAM milling machine (Dental Concept Systems, GmBH, Wesertal, Germany) and the provisional full arch bridge was printed using D-Tech resin in an Asiga printer (Figure 4A, 4B, Video 2). Six months after implant placement, the provisional 3D-printed prosthesis was removed and replaced with a new, durable prosthesis made of G-Cam material.

**Video 2:** Prosthesis fixation

Postoperative outcomes

Immediate rehabilitation with single-piece compressive multi-unit implants using the 3B-TB protocol yielded good aesthetic and functional results at two years postoperative follow-up (Figure 4C). This treatment does not require additional implant support in zones 2 or 3. By applying the biomechanical principles of the 3B-TB protocol, masticatory forces are dampened and distributed before being transferred to the bone implant interface. This reduces excess micro-movements and facilitates immediate loading, safe osseointegration and long-term stability. The patient was postoperatively reviewed for a period of two years and no significant complications were observed.

## Discussion

Management of severely atrophic maxilla poses a significant challenge for clinicians in determining the specific treatment modality. The choice of zygomatic dental implants is of great concern, as it renders successful prosthetic outcomes and, is considered the end option for immediate fixed restoration in atrophic maxillae. However, the preference for zygomatic dental implants can be eliminated by enabling a distinct innovative 3B-TB protocol, which yields excellent outcomes by adhering to the unique mechanical and surgical principles.

**Figure 5:**
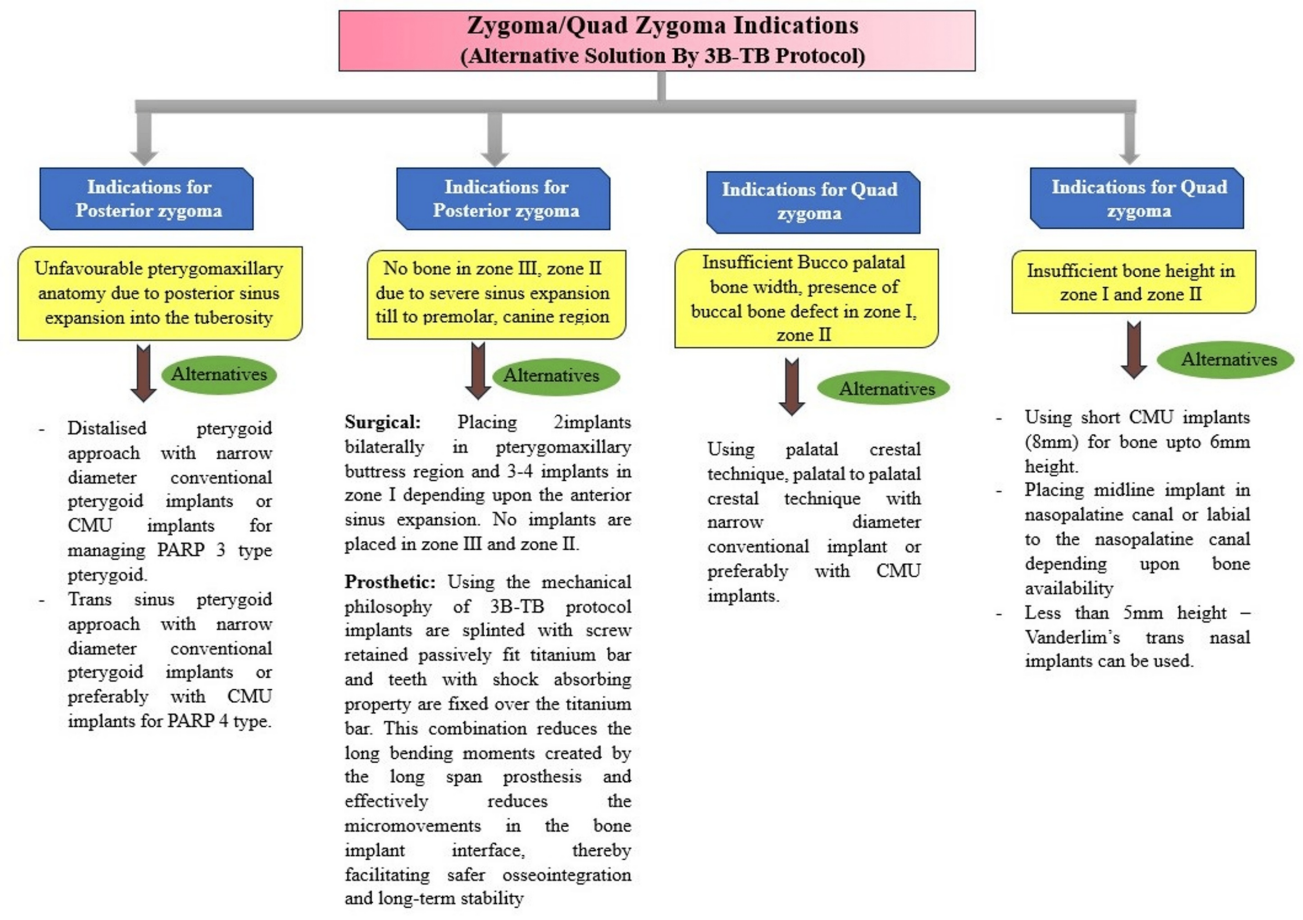
Flowchart showing alternate solutions for zygomatic implant indications using the 3B-TB protocol The images submitted are original and published under a Creative Commons License CMU: Compressive multiunit, PARP: Pterygoid anatomic radiographic prediction

The technical note discusses the indications for zygomatic dental implants, which can be counteracted by dental implant placement and prosthesis using the 3B-TB protocol (Figure 5).

Sinus pneumatization of the maxillary tuberosity, unfavorable for placement of pterygoid implants, can be managed by using trans-sinus pterygoid or distalised pterygoid approaches in the island between the hamular notch and the posterior wall of the sinus. Conventional implants may not be suitable, as there is a high risk of maxillary tuberosity fracture or conditions that could redirect the implant into the maxillary sinus. Hence, single-piece CMU implants would be an ideal choice with an apical diameter of 1.5mm to 2mm and also with their inbuilt multiunit abutment design, which prevents the risk of implants being displaced into the sinus cavity or infratemporal fossa.

Sinus pneumatization in zones 3 and 2 anteriorly is a common indication for zygomatic implants. However, in the 3B-TB protocol, zygomatic implants can be avoided by placing only pterygoid implants and implants in zone 1 and by employing the two key mechanical principles of the 3B-TB protocol. The first principle was to provide teeth with a low elastic modulus for better shock absorption, and the second principle was to use a screw retained passive fit titanium bar for optimal splinting of implants and stress distribution. This combination reduces the excess long bending moments created by the long inter-implant distance and also minimizes excess micromovements at the bone-implant interface.

A lack of bone width and the presence of labial bone defects in zones 3 and 2 are common indications for zygomatic dental implants. These challenges can be addressed in the 3B-TB protocol using the palatal crestal technique and the palatal to palatal crest technique. Even bone thickness of only 1.5-2mm can also be managed with palatal techniques and CMU implants. In conditions of deficient bone in both width and height, short-length (8mm) CMU implants can be used with the palatal technique [14]. Single-piece CMU implants are highly prioritized in 1.5-2mm thin alveolar ridges, due to their unique tapered body and thin apical diameter of 2mm, which restricts the fracture of the buccal plate when compared to conventional implants.

The challenge of reduced bone height of up to 6-8mm in zones 2 and 1 can be mitigated by avoiding zygomatic implants and using short implants of 8mm length, engaging 1-2mm of the implant apex into the nasal floor. In cases of extreme atrophy with only 2-6mm bone height, the alternative option was to place two trans-nasal implants, each on both sides, and one implant in the nasopalatine canal connected to the pterygoid implants [12]. Zygomatic implants in the management of atrophic maxillae predict success and survival rates of >80%. Besides, zygomatic implants elicit numerous complications such as peri-implantitis, periodontitis [15], mucositis, and osseointegration failure [16] postoperatively. Whereas in the present technical report, the patients, when reviewed postoperatively for two years, evidenced no associated complications with the 3B-TB protocol of implant placement. 

The 3B-TB protocol, however, presents a few limitations. This protocol is not indicated for post-maxillary resection defects and post-mucormycosis defects, where there is a lack of existing alveolar bone and basal bone in the maxilla. In these situations, zygomatic implants are essential. In cases of severely failed full arch implants, including pterygoid implants with no remaining alveolar bone, the 3B-TB protocol is not indicated. They can be better treated with quad zygomatic implants. However, if the patient can wait for eight to 10 months of bone healing after removal of failed implants, the 3B-TB protocol can be considered for rehabilitation. Failure to engage the pterygomaxillary buttress during surgery may result in zygomatic implant placement. However, with proper planning and implant selection, the employment of a distalised pterygoid approach and a trans-sinus pterygoid approach can enable implants to be placed successfully in a predictable manner. The patient was postoperatively reviewed for a period of two years only; hence, long-term studies are required to assess the stability and longevity of implants placed using this technique.

## Conclusions

Rehabilitation of atrophic maxilla has been a challenge to clinicians. Zygomatic implants have been proven to be a useful and standard technique for the rehabilitation of atrophic maxilla. However, zygomatic implants are not without complications and failures. Zygomatic implant failures may result in devastating effects on the patients. The options available for clinicians to rehabilitate patients post-zygoma implant failures are very limited, and correction requires a high level of experience. Henceforth, clinicians should look out for ways to avoid zygomatic implants and consider them as a last resort. The 3B-TB protocol could be a feasible and useful alternative to zygomatic implants for the treatment of atrophic maxilla.
